# Intake of Polydextrose Alters Hematology and the Profile of Short Chain Fatty Acids in Partially Gastrectomized Rats

**DOI:** 10.3390/nu10060792

**Published:** 2018-06-20

**Authors:** Mariane Moreira Ramiro do Carmo, Ulana Chaves Sarmento, Leandro Fontoura Cavalheiro, Anderson Fernandes, Wander Fernando de Oliveira Filiú, Karine de Cássia Freitas Gielow, Deiler Sampaio Costa, Adriana Conceiçon Guercio, Valter Aragão do Nascimento, Camila Fontoura Acosta Ribeiro, Alinne Pereira de Castro, Cristiano Marcelo Espinola Carvalho, Daiana Novello, Valfredo de Almeida Santos-Junior, Priscila Neder Morato, Jaime Amaya-Farfan, Priscila Aiko Hiane, Elisvânia Freitas dos Santos

**Affiliations:** 1 Federal University of Mato Grosso do Sul, Campo Grande, 79070-900 Mato Grosso do Sul, Brazil; mariane-nutricionista@hotmail.com (M.M.R.d.C.); ulanachaves@hotmail.com (U.C.S.); lernfc@gmail.com (L.F.C.); andersonbiologo2010@gmail.com (A.F.); wfiliu@terra.com.br (W.F.d.O.F.); kcfreitas@gmail.com (K.d.C.F.G.); deilercosta@gmail.com (D.S.C.); driguercio@yahoo.com.br (A.C.G.); aragao60@hotmail.com (V.A.d.N.); priscila.hiane@ufms.br (P.A.H.); 2 Universidade Católica Dom Bosco, Campo Grande, 79117-900 Mato Grosso do Sul, Brazil; camila.faribeiro@gmail.com (C.F.A.R.); alinne_castro@ucdb.br (A.P.d.C.); cristiano@ucdb.br (C.M.E.C.); 3 State University of Centro-Oeste, Guarapuava, 85040-080 Paraná, Brazil; nutridai@gmail.com; 4 University of Campinas, 13083-862 Campinas, SP, Brazil; juniorfe@gmail.com (V.d.A.S.-J.); jaf@unicamp.br (J.A.-F.); 5 State University of Mato Grosso do Sul, Dourados, 79804-970 Mato Grosso do Sul, Brazil; primorato@gmail.com

**Keywords:** iron-deficiency anemia, gastrectomy, prebiotics, rats

## Abstract

Polydextrose (PDX) ingestion may increase the intestinal absorption of iron. This study evaluated the effects of 7.5% polydextrose supplementation on markers of iron uptake, transport and storage in partially gastrectomized rats. Half of a batch of 40 male Wistar rats (250 g) underwent Billroth II partial gastrectomy with anterior truncal vagotomy (GXT), while the other half underwent sham gastrectomy (SHAM). At 7 postoperative days, the animals were subdivided into four groups (*n* = 10): Sham Control and GXT Control (no polydextrose); Sham PDX and GXT PDX (with 7.5% PDX). The animals were euthanized after 60 day of PDX treatment. Organ weight, cecal pH, the characterization and quantification of short-chain fatty acids (SCFA), hematological parameters, hepatic iron content and the expression of ferroportin (FPT) in the jejunum, cecum, colon and liver were evaluated. PDX caused changes in the cecum of the supplemented animals, where there was a decrease in pH, increase in cecal wall and marked production of SCFA, especially acetic and propionic acids (*p* < 0.05). Hepatic iron levels were lower in GXT animals. PDX increased hemoglobin (HGB) values by 29.2% and hematocrit (HCT) by 55.8% in the GXT PDX group compared to the GXT Control group. The GXT PDX group had lower hepatic FPT expression (*p* < 0.05). PDX led to increased SCFA concentration in the supplemented animals. Considering that SCFAs play a central role in the increasing nutrients uptake, this mechanism may be involved in altering the hematology profile observed in these animals but not enough to reverse iron deficiency anemia in post-gastrectomy rats.

## 1. Introduction

Gastric cancer is characterized as the disordered growth of cells in stomach tissue [1]. Stomachal tumors are now the fifth most common cancer in the world, representing 6.8% of all carcinoma diagnoses, which is the third most common cause of death due to malignant neoplasms [2]. The main form of curative treatment of gastric cancer is surgical resection, most commonly partial or total gastrectomy [1,3,4].

Iron-deficiency anemia is common after some gastrointestinal surgeries, such as gastric resections (gastrectomies), affecting approximately 60% of gastrectomized patients [5,6]. Iron deficiency in oncological patients combined with gastrointestinal complications compromise the efficacy of chemotherapy, promote general malnutrition, aggravate cancer cachexia, and may contribute to a worse prognosis [7]. Therefore, treating iron deficiency anemia becomes essential to improve the quality and life expectancy of these individuals. Iron is normally absorbed in the small intestine and the stomach plays the lead role in improving the biological availability of this mineral [8,9,10]. However, when its absorption pathway is not sufficient to fulfill the needs of the body, iron can also be absorbed from the colon [11,12].

Evidences point out several benefits in iron absorption from the ingestion of prebiotics [13,14,15]. Polydextrose (PDX), a highly branched and randomly linked glucose polymer with a mean degree of polymerization of 12, ranging from 2 to 120 and containing all possible combinations of 1→2, 1→3, 1→4 and 1→6 glycosidic bonds, with 1→6 (both α and β) predominating, has received special attention in iron metabolism and absorption [10,16,17,18,19]. PDX is not hydrolyzed by mammalian digestive enzymes in the small intestine, going intact into the colon. In the large intestine, its fermentation is gradual and slow, resulting in a great production of short-chain fatty acids (SCFA) altering the composition of the microbiota [12,20,21].

Among the most known SCFAs, stand acetate, butyrate, propionate and lactate. The resulting SCFAs from the fermentation of PDX act on the reduction of intestinal pH and, as a consequence, they make the growth of pathogenic bacteria difficult (*Salmonella* spp., and *Escherichia coli*) and facilitate the absorption of minerals (especially calcium, zinc and iron). In parallel, they serve as a substrate for colonocytes (particularly butyrate), stimulating the growth and/or the activity of beneficial bacterias (*Lactobacillus* and *Bifidobacteria*) [22,23,24,25,26]. Such benefits are important for the health maintenance of patients recovering from gastric surgeries [27,28].

The SCFAs produced during the fermentation, especially the propionic acid, seem to play a role in the increase of iron absorption, forming soluble complexes with the mineral facilitating its absorption [26]. This increased absorption is also due to the increased absorption area, mainly of the cecal tissue. When this area is increased, consequently, the number of iron receptors present in the colon increases, with a greater expression of brush border enzymes and the nutrient transport system [29,30,31]. In addition, changes in the architecture of the intestinal mucosa, as a result of increases in both cellularity and number of crypts, are factors that may contribute to an increase in the surface of mineral absorption [32].

In a postmenopausal study model in rodents, PDX consumption has been shown to lead to the increased production of SCFAs and increased absorption of minerals (calcium and magnesium) in the colon [33]. Santos et al. [10] and Santos et al. [34] concluded that PDX supplementation increased the uptake of calcium [10] and iron [34] in partially gastrectomized rats. In another study performed on fully gastrectomized rats fed a diet rich in PDX, the authors observed higher Ca uptake and bone mineralization, increased tissue and cecal content and reduced cecal pH in the PDX-supplemented groups when compared to groups receiving a basal diet and/or partially hydrolyzed guar gum (GGPH) [35].

In this context, prebiotics have been proposed as therapeutic factors. Although it is widely accepted that prebiotics help balance the intestinal microbiota [36], its effects on iron uptake and the mechanisms involved are poorly understood. A better understanding of the prebiotic potential of PDX and the action mechanisms involved in the increasing iron uptake would be useful in the planning of diets with greater bioavailability of this mineral [36,37]. Therefore, PDX supplementation may be an alternative nutritional support for increased intestinal absorption of iron in patients submitted to gastric surgery. Thus, the goal of the present study was to evaluate the effects of 7.5% PDX supplementation on iron metabolism and on the short-chain fatty acid profile in partially gastrectomized rats.

## 2. Materials and Methods

### 2.1. Animals and Experimental Conditions

Male Wistar rats (*Rattus norvergicus* Wistar) (*n* = 40) of about 30 days of age, weighing around 100 g, were obtained from the colonies kept in the Central Animal House of the Federal University of Mato Grosso do Sul (UFMS), Mato Grosso do Sul, Brazil. The animals were kept in collective cages throughout the experimental period, except on days of blood collection, in an environment with controlled temperature (23 ± 2 °C) and 55% relative humidity, with a dark-light cycle of 12 h starting at 7:00 h.

For growth up to 200 g, the animals were fed commercial, unpurified and closed-label commercial BioBase feed (BioTec^®^ Lot: 20112014, Águas Frias, Santa Catarina, Brazil). After attaining this weight, the animals were fed standard AIN-93M feed [38] until they reached the ideal weight for the surgical procedure (approximately 250 g). Water and feed were offered ad libitum. The entire experiment was carried out in accordance with international ethical principles and procedures and approved by the UFMS Ethics Committee on Animal Use under protocol No. 640/2014.

### 2.2. Surgical Procedure

The surgical procedure was performed strictly following the principles of the specific surgical technique already standardized, and according to the anatomy of the animal. The animals (mean weight of 250 g) were randomly divided into two groups: gastrectomized (GXT) (*n* = 20) and sham operated (Sham) (*n* = 20). For surgery, the rats were fasted for approximately 8 h and previously anesthetized with combined ketamine (80 mg/kg) and xylazine (10 mg/kg), intraperitoneally. Anesthesia was checked by the absence of neuromuscular reflex. For the development of iron-deficiency anemia, the animals of the GXT group underwent Billroth II gastrectomy with anterior truncal vagotomy, a gastro-enteroanastomosis (stomach/jejunum) for reconstruction of the traffic [39]. The Sham group (*n* = 20) underwent the same surgical stress, the abdominal cavity was held open for approximately 45 min (estimated duration of gastrectomy) in order to promote surgical stress similar to that of GXT animals.

### 2.3. Postoperative Care

Immediately after the end of the surgery, the animals were placed next to a heater (Britânia Fama-Model AQF 1000N, Curitiba, Paraná, Brazil), until complete reestablishment of the anesthesia. The rats were given orally, ad libitum, 5% glycosylated serum (KabiPac^®^, Fresenius Kabi Brazil Ltda Lot 744L3879, Aquiraz, Ceará, Brazil) for 12 h, and they then had free access to AIN93-M and water [10].

During the first 5 postoperative days, the animals received intramuscular injections of an antiinflammatory agent with analgesic properties (Meloxicam, 0.1 mg/kg) (Eurofarma^®^, São Paulo, SP, Brazil), once a day.

### 2.4. Experimental Design

At 7 days after the surgical procedure, the animals were separated into four groups of 10 animals, which were designated as follows: Sham Control group: sham animals given AIN-93M feed without addition of polydextrose; Sham PDX group: sham animals given AIN-93M feed with addition of 7.5% polydextrose; GXT Control group: gastrectomized animals given AIN-93M feed without addition of polydextrose; and GXT PDX group: gastrectomized animals given AIN-93M ration with addition of 7.5% polydextrose.

**Figure 1:**
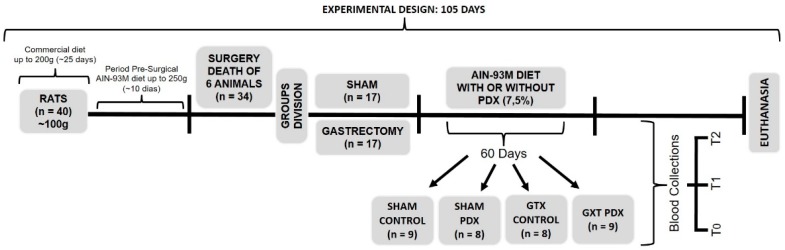
Experimental design. T0 (Time 0): 7 days after the surgical procedure; T1 (Time 1): after 1 month on experimental diet; T2 (Time 2): after 2 months on experimental diet (before euthanasia).

Of the 40 rats, six died after the surgical procedure, three of the GXT group and three of the Sham group (according to Figure 1).

### 2.5. Experimental Diets

Supplementation was started 7 days after the surgical procedure and had a duration of 60 days. Two types of diets were prepared: Standard AIN-93M control diet, without addition of polydextrose (PDX) and supplemented diet, with addition of PDX at 7.5/100 g of feed, both prepared according with the “American Institute of Nutrition” [38]. PDX was added to the standard formulation of the AIN-93M feed in place of cellulose and part of the corn starch as described in Table 1. Experimental diets were pelleted, rats consumed their respective diets and water was provided ad libitum except on the days of blood collection.

After the beginning of the administration of the different diets, feed consumption and weight gain of the animals were monitored twice a week. Measurement of dietary intake was performed considering the amount of feed offered in each cage and subtracting the leftovers. The result was divided by the number of rats housed in the respective cages and number of days. The weight gain of the animals was performed individually with the rats placed in a plastic container. The feed and animals were weighed on a semi-analytical digital balance (Marte Científica & Instrumentação Industrial Ltda, São Paulo, SP, Brazil, model LS2), with maximum capacity of 2010 g and accuracy of 0.5 g. At the end of the 60-day supplementation period, the feed efficiency coefficient (FEC) was calculated by dividing the weight gain (g) by dietary intake (g) [40].

### 2.6. Supplementation of Vitamin B12 (Cyanocobalamin)

One week after surgery, gastrectomized rats began to receive vitamin B12 supplementation (Bedoze^®^ SM Register: 014/75, São Paulo, SP, Brazil). This was given intramuscularly at a dose of 0.5 mg/kg every 15 days until the end of the experiment [41,42], to prevent the development of megaloblastic anemia. The animals of the Sham group received 0.9% sodium chloride (Arboreto^®^ Ltda.-Lot: 15125715, Juiz de Fora, MG, Brazil) to simulate the same stress.

### 2.7. Euthanasia

Euthanasia was performed 60 days after the start of supplementation of PDX. For euthanasia the animals were fasted for 8 h. The rats were anesthetized intraperitoneally with a combination of ketamine (80 mg/kg) and xylazine (10 mg/kg). Body length (cm) was then measured with a tape measure [40], and soon after, blood was collected by cardiac puncture. After blood collection, euthanasia was confirmed in a CO_2_ chamber. With the animals euthanized, we collected the organs and tissues for further analysis.

The collected organs and tissues (jejunum, cecum, colon and liver) were weighed on a semi-analytical electronic balance (Marte^®^, model: AD 5005, Refer.: 124.0500.02, São Paulo, SP, Brazil) and the weight is expressed in grams. The cecum was weighed after removal of the cecal contents.

### 2.8. Measurement of pH of Cecal Contents

After confirmation of euthanasia, the abdominal cavity of the animals was opened and the cecum located. The pH of the cecal contents was measured through a small incision in the wall of the organ, where a 5 mm diameter portable electrode (Digimed Instrumentação Analítica, DM-22, São Paulo, SP, Brazil) was introduced. For this, the animals were sacrificed between 6:00 a.m. and 10:00 a.m., a period in which intestinal fermentation is more intense [30,43].

### 2.9. Analytical Methods

#### 2.9.1. Percentage Composition of Formulated Diets

The total nitrogen and protein content was determined by the micro-Kjeldahl method, according to the method described elsewhere [44]. Total lipids were obtained by gravimetry, according to the method described by Bligh and Dyer [45]. Moisture, total solids and ash were determined by gravimetry, according to the procedures described elsewhere [44]. The total carbohydrate content was obtained by theoretical calculation (by difference) in the results of triplicates, according to the formula: % carbohydrates = 100 − (% moisture + % protein + % lipids + % ash). The caloric value was determined by the sum of crude protein, lipids and total carbohydrates, according to the formula: Caloric value = (crude protein × 4) + (total lipids × 9) + (carbohydrates × 4).

#### 2.9.2. Determination of Iron Content in Diets and Liver Samples

The diets were stored in a freezer at −18 °C (Eletrolux^®^, Air Flow System Model: DC48, São Carlos, SP, Brazil), and at the end of the experiment, they were ground in a mill (Tecnal^®^ Equipamentos para Laboratórios Ltda., Model: TE-631/1, Piracicaba, SP, Brazil) for further analysis. The liver, after collection, was washed with saline, gently dried on filter paper, weighed and then stored in a freezer at −18 °C (Eletrolux^®^). At the time of analysis, the liver was thawed and the analysis was performed on a wet basis. For determination of iron, 0.5 g of each sample was weighed out. Each aliquot was placed in a DAP 60 tube, suitable for microwave digestion (Speedwave^®^ Berghof, Germany), together with 5 mL of 65% nitric acid (Merck^®^, Rio de Janeiro, Brazil) and 3 mL of 35% hydrogen peroxide (Merck). After digestion, ultrapure water (PURELAB Option-Q^®^, Elga-Veolia, UK) was added to a final volume of 100 mL. The sample readings were performed with an inductively coupled argon-plasma optical emission spectrometer (ICP-OES-Icap 6000^®^ Thermo Scientific, Waltham, Massachusetts, USA). High-purity argon gas (99.999%) and standard multi-element stock solution containing 100 mg/L iron (Aldrich^®^, Milwaukee, WI, USA) were used. The ICP-EOS was optimized for a wavelength of 238.2 nm [46].

#### 2.9.3. Hematocrit, Hemoglobin, Mean Corpuscular Volume and Mean Corpuscular Hemoglobin Concentration

With the animals anesthetized, blood was collected with a heparinized capillary tube (Vitrex Medical^®^, Model: DK-2730, Ref: 161315, Lancashire, England, UK,) from the retro-orbital plexus. Hematology microtubes with K_3_-EDTA (Vacatube Micro^®^, Model: GD005EK, Ref: 120423, Franklin, Tennessee, USA) were used. Blood samples were collected at three times during the study: Time 0: 7 days after the surgical procedure; time 1: after 1 month on experimental diet, and time 2: after 2 months on experimental diet (before euthanasia). On the day before collection, the animals were housed in individual cages and fasted for 8 h. Hematocrit, hemoglobin and RBC indices were determined in an automated analyzer (Roche Hitache, Model Cobas 6000, Tokyo, Japan).

#### 2.9.4. Collection of Blood Samples for Serum Iron Parameters

The animals were housed in individual cages and fasted for 8 h. Blood was collected after the animals were anesthetized (in Vacuette^®^ tubes with clot activator and serum-separating gel, Model: 8019895, Franklin, Tennessee, USA). The blood was then centrifuged at 7000 rpm for 15 min in a centrifuge (Fanem^®^, Model: 206/1, São Paulo, SP, Brazil) to obtain serum, which was used to determine all serum iron parameters. Serum levels of iron and unsaturated iron binding capacity (UIBC) were measured using an automated analyzer (Roche^®^ Hitache brand, Model Cobas 6000, Tokyo, Japan). Total iron binding capacity (TIBC) was obtained by the equation: TIBC = serum iron + UIBC. Transferrin saturation index (TSI) was calculated by the equation: TSI = serum iron/TIBC × 100 [39].

#### 2.9.5. Protein Analysis by Western Blotting

The tissues (jejunum, cecum, colon and liver) were homogenized in an extraction buffer (200 mmol/L EDTA, 1 mol/L Tris Base, 10 mmol/L orthovanadate, 2 mmol/L phenylmethanesulfonyl fluoride, 10 mmol/L sodium pyrophosphate, 0.1 mg/mL aprotinin, 100 mmol/L sodium fluoride, Triton 10%, ultrapure water) using Polytron (Pro Scientific model Pro 200, Oxford, CT, USA). After homogenization, 10% triton X-100 was added to the samples, and these were kept on ice for 30 min and centrifuged at 15,000 rpm/40 min at 4 °C (Beckman, Fullerton, CA, USA). The supernatants were collected and the levels of total protein were determined by the Lowry et al. [47] method. The samples were treated with Laemmli buffer (0.01% Bromophenol blue, 50 mM sodium phosphate, Glycerol 25%, SDS 1%) containing dithiothreitol (200 mM).

The samples were applied to an 8% SDS-PAGE and transferred using a semidry system (Bio-Rad, Hercules, CA, USA) to a nitrocellulose membrane (0.22 μm pore size Santa Cruz, CA, USA). The membranes were blocked at 4 °C, for 30 min, with 5 mL of blocking buffer, baseline solution (Trizma base 100 mM, St. Louis, MO, USA; EDTA mM; Triton X-100 0.5%; sodium orthovanadate 2 mM), and 3% bovine albumin. The nitrocellulose membranes were incubated overnight at 4 °C with a Ferroportin 1 antibody (ferroportin), with a molecular weight of 68 kDa, using Abcam (Cambridge, MA, USA, catalogue number ab58695), in a 1:1000 dilution in a blocking buffer. The appropriate secondary antibodies were used for detection.

Subsequently, the membranes were visualized with a UVITEC Cambridge instrument (model Alliance LD2, Rugby, Warwickshire, UK). The intensity of the bands of interest was identified by the pattern of electrophorectic motility and quantified by optical densitometry using the digital program Image J (v1.51 for Windows, NIH, Bethesda, MD, USA).

#### 2.9.6. Collection of Cecal Contents of Animals

After confirmation of euthanasia, the abdominal cavity of the animals was opened and the cecum marked and removed. The contents of the cecum were then collected in 2-mL Eppendorf^®^ tubes (Kasvi^®^, Model: K6-0200, Lot: 78293, São José do Pinhais, PR, Brazil) and immediately frozen at −18 °C for further quantification of SCFA.

#### 2.9.7. Characterization and Quantification of Fatty Acids

SCFA were extracted from the cecum contents according to Zhao et al. [48], with modifications. A half gram of cecum contents was added to 5 mL of water and the mixture homogenized for 3 min. The pH of the cecal suspension was adjusted to 2 to 3 by the addition of 5M HCl solution. This mixture was then transferred to polypropylene tubes and centrifuged for 30 min at 3000 rpm (Model: 206/1 Centrifuge; Fanem São Paulo, Brazil), afterwards, the supernatant obtained was filtered with a 0.45-μm microfilter, obtaining a clean extract that was injected in a gas chromatograph for quantification of SCFA.

The chromatograph (Shimadzu Scientific GC 2010 Kyoto, Honshu, Japan) was equipped with a flame ionization detector. A NUKOL column, 30 m × 0.53 mm (internal diameter) × 0.50 μm (film thickness), was heated using the following program: isothermal at 165 °C for 5 min, 10 °C/min up to 195 °C, and maintained isothermally at 195 °C for 4 min. The other chromatographic conditions were: injected sample volume of 1 μL, injector temperature of 230 °C), detector temperature of 230 °C), split ratio of 15, drag gas flow rate (He) of 1.97 mL/min; make-up flow or auxiliary gas (N_2_) flow rate of 30 mL/min; flue gas flow rate: synthetic air (400 mL/min) and H_2_ (40 mL/min).

#### 2.9.8. Statistical Analyzes

Data were analyzed using GraphPad Prism software, version 6.01 (GraphPad Software, La Jolla, CA, USA). All data showed normal distribution and were submitted to analysis of variance (ANOVA), followed by Tukey’s post-hoc test (Tukey’s multiple comparison test) *p* < 0.05. Results were expressed as mean ± standard error of mean (SEM) or standard deviation (SD).

## 3. Results

### 3.1. Composition of Experimental Diets

Experimental diets showed statistical differences between them in relation to ash content (*p* = 0.0002), but did not show statistically significant differences (*p* > 0.05) in relation to the macronutrient and iron contents, being considered isoenergetic and isoproteic.

In this context, these results are shown in Table 2.

### 3.2. Dietary Intake, Weight Gain and Feed Efficiency Coefficient (FEC)

The dietary intake, body weight, body length and FEC of the animals are shown in Table 3. The animals of the Sham Control group showed higher dietary intake (*p* < 0.0001) when compared to the other experimental groups. The GXT PDX group had the lowest dietary intake (1008.88 ± 5.81 g), but when the GXT Control group was compared to the Sham PDX group, there was no significant difference between (*p* = 0.7350). The GXT Control group had a lower weight gain than the Sham Control (*p* = 0.0075) and Sham PDX (*p* = 0.0315) groups, but when compared to the GXT PDX group, there was no significant difference (*p* = 0.4727). There was no statistical difference in weight gain between the GXT PDX group and Sham PDX (*p* = 0.5257) and Sham Control (*p* = 0.2146) groups. Accordingly, it was possible to see that the GXT (Control and PDX) groups exhibited lower FE, differing statistically from the Sham Control (*p* = 0.0104) and Sham PDX groups (*p* = 0.0104). Regarding body length of the animals, there was no statistical difference between the experimental groups (*p* > 0.05).

### 3.3. Fermentation Parameters

#### 3.3.1. Cecal Wall Weight

**Figure 2:**
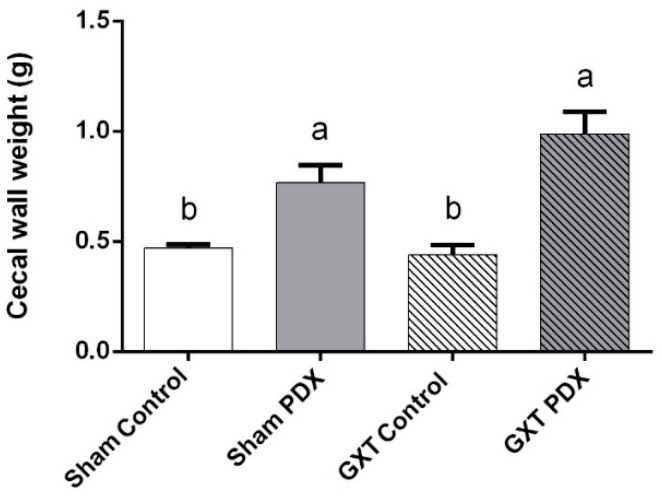
Cecal wall weight (g) of the animals of the experimental groups. Bars with same letters indicate no significant difference according to Tukey’s test (*p* < 0.05). Different letters indicate a significant difference according to Tukey’s test (*p* < 0.05). Sham Control: sham without polydextrose; Sham PDX: sham with polydextrose (7.5%); GXT Control: gastrectomized without polydextrose; GXT PDX: Ggastrectomized with polydextrose (7.5%). Values expressed as mean ± SEM.

There was a significant increase in the cecal weight of animals that received polydextrose (Sham PDX and GXT PDX), in relation to the Sham Control group (*p* = 0.0195 and *p* = 0.0001, respectively) and GXT Control group (*p* = 0.0090 and *p* = 0.0001, respectively). The GXT PDX group had a 41% higher mean cecal wall weight compared to the GXT Control group as shown in Figure 2.

#### 3.3.2. pH of Cecal Contents

**Figure 3:**
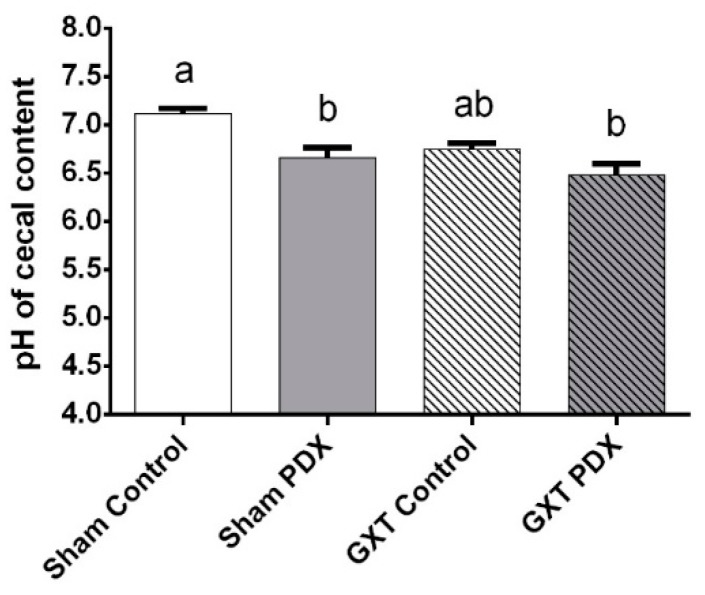
pH of cecal contents of animals of the experimental groups. Bars with same letters indicate no significant difference according to Tukey’s test (*p* < 0.05). Different letters indicate a significant difference according to Tukey’s test (*p* < 0.05). Sham Control: sham without polydextrose; Sham PDX: sham with polydextrose (7.5%); GXT Control: gastrectomized without polydextrose; GXT PDX: gastrectomized with polydextrose (7.5%). Values expressed as mean ± SEM.

The pH values of the cecal contents of the animals are shown in Figure 3. The Sham Control group had the highest mean cecal pH, differing statistically from the Sham PDX (*p* = 0.0093) and GXT PDX (*p* = 0.0004) groups. The groups that received PDX (Sham PDX and GXT PDX) had a lower mean pH (6.66 ± 3.31 and 6.48 ± 3.16, respectively). The pH cecal contents of the GXT PDX group indicated substantially more acid than that of the Sham Control group.

#### 3.3.3. Characterization and Quantification of Short-Chain Fatty Acids (SCFA)

**Figure 4:**
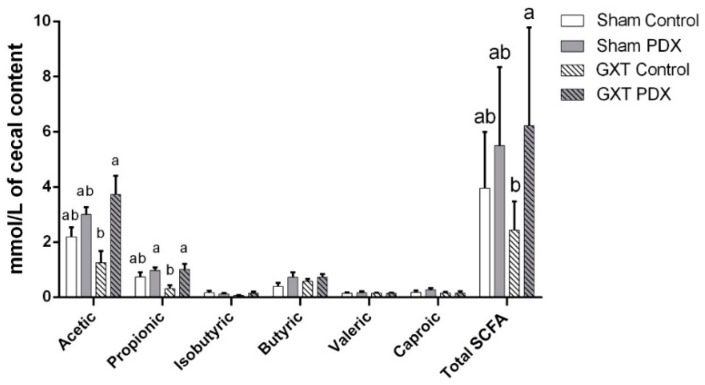
Concentration of SCFA (mmol/L) (acetic acid, propionic acid, isobutyl acid, butyric acid, valeric acid, caproic acid and total SCFA) of animals in the experimental group. Bars with same letters indicate no significant difference according to Tukey’s test (*p* < 0.05). Different letters indicate a significant difference to the Tukey test (*p* < 0.05). Sham Control: sham without polydextrose; Sham PDX: sham with polydextrose (7.5%); GXT Control: gastrectomized without polydextrose; GXT PDX: gastrectomized with polydextrose (7.5%). Values expressed as mean ± SEM.

Figure 4 shows the characterization and quantification of SCFA. The GXT PDX group had a mean acetic acid that was 198.4% higher than that of the GXT Control group (*p* = 0.0030). As for propionic acid, the groups supplemented with PDX (Sham PDX and GXT PDX) showed higher means, differing statistically from the GXT Control group (*p* = 0.0277 and *p* = 0.0171, respectively). The GXT PDX group had 215.6% higher concentration of this fatty acid when compared to the GXT Control group. It is also evident that there were significant differences with regard to total SCFA between the experimental groups. In this evaluation, the GXT PDX group showed a 156% higher concentration than the GXT Control group (*p* = 0.0403), but there was no statistical difference between the GXT PDX group and Sham Control (*p* = 1.462) and Sham PDX (*p* = 1.418) groups.

### 3.4. Hematologic Parameters

#### 3.4.1. Hemoglobin (HGB), Hematocrit (HCT), Mean Corpuscular Volume (MCV) and Mean Corpuscular Hemoglobin Concentration (MCHC)

**Figure 5:**
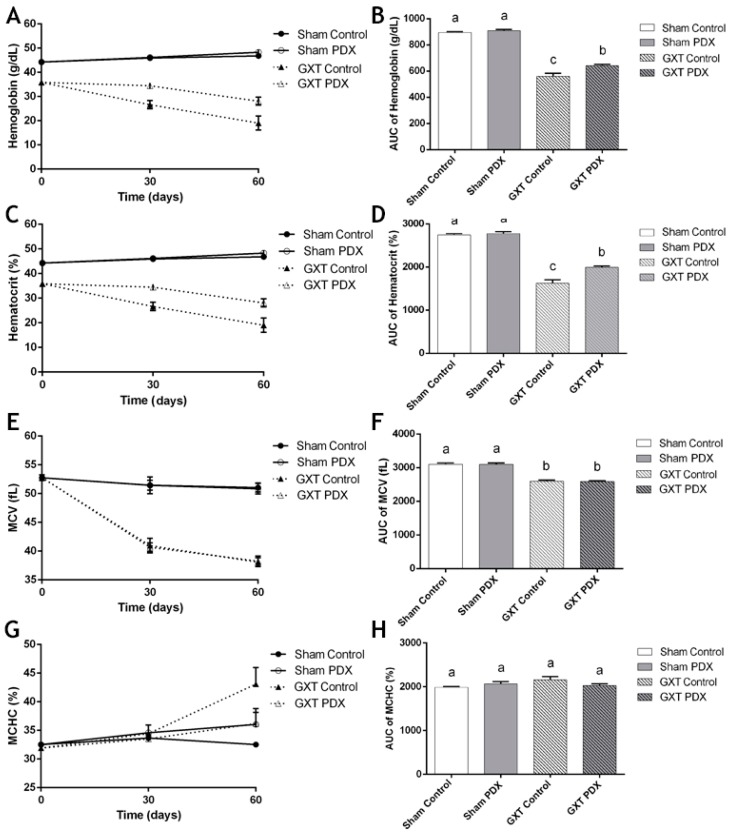
Hematologic parameters of experimental groups. Hemoglobin (g/dL) concentrations at different times (**A**) and area under the curve (**B**). Hematocrit (%) levels at different times (**C**) and area under the curve (**D**). MCV (fL) levels at different times (**E**) and area under the curve (**F**). MCHC (%) levels at different times (**G**) and area under the curve (**H**). Absence of letters and bars with same letters indicate no significant difference according to Tukey’s test (*p* < 0.05). Different letters indicate a significant difference according to Tukey’s test (*p* < 0.05). Sham Control: sham without polydextrose; Sham PDX: sham with polydextrose (7.5%); GXT Control: gastrectomized without polydextrose; GXT PDX: gastrectomized with polydextrose (7.5%). Values expressed as mean ± SEM.

The concentration and area under the curve values of HGB (g/dL), HCT (%), MCV (fL) and MCHC (%) are shown in Figure 5A–H, respectively, at the times evaluated during the supplementation period. The GXT groups (Control and PDX) showed a marked decline in HGB, HCT and MCV shortly after surgery (time 0) (Figure 5A,C). The animals of the Sham (groups Control and PDX) had statistically higher HGB and HCT levels than the GXT groups (Control and PDX) (*p* < 0.0001). However, the areas under the curve for HGB and HCT of the animals of the GXT PDX group (Figure 5B,D) were respectively 29.2% and 55.8% higher compared to the GXT Control group animals (*p* = 0.0016 and *p* < 0.0001, respectively). It was also observed that the mean values of the area under the curve for MCV did not differ between the Sham Control and Sham PDX groups (*p* = 0.9998). The same lack of significant difference was seen between the GXT Control and GXT PDX groups (*p* = 0.9999). However, when comparing animals from the GXT groups to the Sham groups, the GXT Control and GXT PDX groups had markedly lower MCV levels compared to the Sham Control and Sham PDX groups (*p* < 0.0001). There were no differences in MCHC (*p* > 0.05) between any of the experimental groups during the 4 weeks of treatment (Figure 5C,D).

#### 3.4.2. Serum Iron Parameters

Serum iron, UIBC, TIBC and TSI (Table 4) showed differences when comparing the Sham groups (Control and PDX) to the GXT groups (Control and PDX) (*p* < 0.0001). No statistical difference was detected between the Sham Control and Sham PDX groups or between the GXT Control and GXT PDX groups (*p* > 0.05).

### 3.5. Reserve Parameters

**Figure 6:**
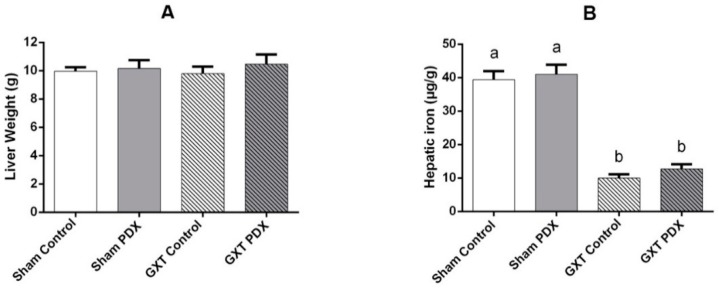
**A**: Liver weight (g) of the experimental groups. **B**: Iron concentrations in the liver (mg/g liver) of the experimental groups. Absence of letters and bars with same letters indicate no significant difference according to Tukey’s test (*p* < 0.05). Different letters indicate a significant difference to the Tukey test (*p* < 0.05). Sham Control: sham without polydextrose; Sham PDX: sham with polydextrose (7.5%); GXT Control: gastrectomized without polydextrose; GXT PDX: gastrectomized with polydextrose (7.5%). Values expressed as mean ± SEM.

Fresh liver weights and hepatic iron levels of animals are shown in Figure 6A,B, respectively. There was no difference between the experimental groups regarding liver weight (*p* > 0.05). The Sham groups (Control and PDX) had a statistically higher mean hepatic iron concentration in relation to the GXT groups (Control and PDX) (*p* < 0.0001).

#### Ferroportin Analysis

**Figure 7:**
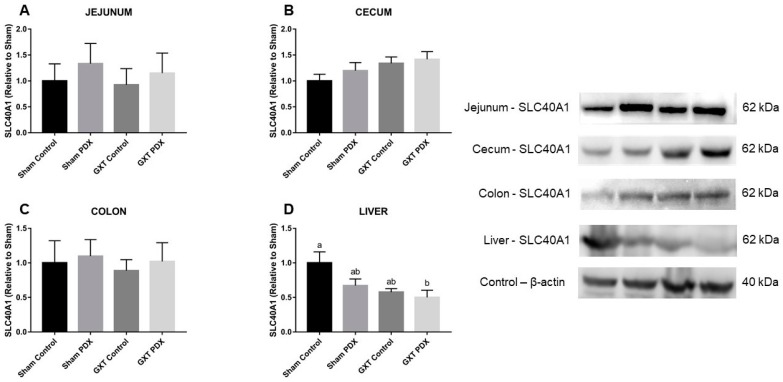
Expression of ferroportin in the jejunum (**A**), cecum (**B**), colon (**C**) and liver (**D**) of the animals of the experimental groups. Different letters indicate a significant difference according to Tukey’s test (*p* < 0.05). Sham Control: sham without polydextrose; Sham PDX: sham with polydextrose (7.5%); GXT Control: gastrectomized without polydextrose; GXT PDX: gastrectomized with polydextrose (7.5%). Values expressed as mean ± SEM.

Ferroportin (FPT) expression values in the animals’ jejunum, cecum, colon and liver are shown in Figure 7A,B, respectively. Western blot analysis did not detect a difference (*p* > 0.05) between the experimental groups with regard to FPT expression in the jejunum, cecum, and colon of the animals but the GXT PDX group showed a trend towards greater FPT expression in the cecum. In relation to FPT expression in the liver (Figure 7B), the GXT PDX group showed the lowest concentration, differing significantly from the Sham Control group (*p* = 0.0482).

## 4. Discussion

After gastrectomy, gastric capacity is reduced, intestinal transit becomes faster and there is a decrease in the site of absorption of the nutrients [49]. Therefore, the lower dietary intake in the GXT Control (*p* = 0.0018) and GXT PDX (*p* < 0.0001) groups compared to Sham Control may be associated with the partial removal of the stomach, and also for the lower dietary intake of the GXT PDX group compared to the Sham PDX group (*p* = 0.0016). In addition, the Sham PDX group had a lower dietary intake than the Sham Control group (*p* < 0.0001). These results, as well as the significant difference of FEC between the GXT PDX group and the Sham groups (Control and PDX) (*p* = 0.0104), can be attributed to the satietogenic effect of PDX, since these animals showed lower dietary intake but similar weight gain as the sham-operated groups.

The mechanisms by which fiber affects satiety are not fully understood [50]. To the best of our knowledge, there have been no animal model studies on the effect of PDX consumption on appetite; however, some studies in humans have shown a positive relationship between PDX and satiety [50,51,52,53,54]. In a meta-analysis, Ibarra et al. [54] showed that PDX promotes a reduction in the subjective feeling of appetite during the satiety period shortly after a meal, results that support the findings are reported here. Another hypothesis that may explain the lower dietary intake of animals receiving PDX (Sham PDX and GXT PDX) than the Sham Control group is related to the modulation of gastrointestinal peptides involved in the regulation of food intake, which are produced by L type enteroendocrine cells [55]. It is believed that the fermentation of prebiotics by colon bacteria stimulates the expression of genes leading to the secretion of different peptides, such as: peptide tyrosine-tyrosine (PYY), cholecystokinin (CCK), glucagon-like peptide-1 (GLP-1) and glucagon-like peptide-2 (GLP-2) by L type enteroendocrine cells [56,57]. All these hormones present themselves as potential biomarkers for satiety. Ghrelin is also a hormone that appears to contribute to appetite modulation, and prebiotics seem to decrease hunger by reducing levels of ghrelin [58,59]. Thus, after a meal rich in prebiotics, hormones such as GLP-1 are released by the intestine, stimulating the feeling of fullness (satiety) [56,57].

The finding that the GXT PDX group achieved a weight gain similar to that of the Sham groups (Control and PDX) even though it had a lower feed intake, can be attributed to the prebiotic action of PDX, where colonic fermentation in the intestines of these animals may have led to modulation of the intestinal microbiota and increased nutrient uptake sites, minimizing the desorption and possible malnutrition caused by partial gastrectomy along with anterior truncal vagotomy. It is also possible that, as in the case with other types of fiber [60], PDX influenced the absorption of macronutrients, especially carbohydrates, delaying gastric emptying and/or decreasing transit time in the large intestine. Additionally, gluconeogenesis may have been induced by PDX supplementation and mediated by SCFA, especially propionic acid, which is in accordance with the results presented in the present study [19,20,21,60,61].

The lower weight gain shown by the animals of the GXT control group, even with a diet similar to that of the Sham PDX group, may be related to the anemia, malnutrition and possible dysbiosis picture caused by gastric resection surgery. Zhang et al. [62] stated that after gastrectomy, complications related to the intestinal microbiota are frequent and include a lower secretion of gastric acid, which may favor bacterial hyperproliferation, intestinal dysmotility, and displacement of bacteria typical of the small intestine into the large intestine.

Another hypothesis that can elucidate the lower FEC in the GXT animals (Control and PDX) in relation to Sham animals (Control and PDX) is the fact that, due to the surgical intervention, these animals were subjected to great stress, which can generate behavioral changes such as changes in dietary patterns (water and food intake) [63,64].

There was no difference (*p* > 0.05) in relation to the total length of the animals between the experimental groups, which indicated that gastric resection surgery with anterior truncal vagotomy and PDX supplementation led to metabolic modifications and, body composition, without influencing growth.

Due to the complex structure and branching of the PDX molecule, its fermentation occurs gradually and slowly in the colon, suggesting that PDX remains available as a carbon source for gut bacteria; this process induces changes in the microbiota and results in the production of SCFA and small amounts of gas [20,61,65,66]. We observed a marked increase in SCFA in cecal contents (198.4% of acetic acid, 215.6% of propionic acid and 156% of total SCFA) and consequent acidification (5 times), causing thickening of the cecal wall (41%) in the supplemented animals.

In the literature, studies indicate that the use of prebiotics may stimulate hypertrophy of the gastrointestinal tract. Other studies have correlated the effect of non-digestible carbohydrates on intestinal mucosal proliferation and maintenance of tissue integrity [15,67,68,69,70]. Such a benefit is extremely important for patients recovering from gastric surgeries [27,28]. The changes promoted from the fermentation of PDX originate from the microbial degradation and consequent use of these degradation byproducts; such as SCFAs, which are consumed as energy sources by cells of the intestinal epithelium (butyric acid), or metabolized by muscle cells (acetic acid) or hepatocytes (propionic acid) [19].

Yoshioka; Shimomura and Suzuki [71] evaluated the effects of different concentrations of PDX on the morphology of the large intestine of rats. The authors observed that PDX caused significant growth of the cecal mucosa compared to the groups that received cellulose as a source of fiber. Costabile et al. [68] in a study with humans, whose main objective was to identify the microbial groups affected by the fermentation of PDX (8 g/day) in the colon, observed after 3 weeks a significant increase in the number of *Ruminococcus intestinalis*, known as the main butyrate producer. The finding that butyric acid concentration was not higher in the experimental groups supplemented with PDX (Sham PDX and GXT PDX) could have been due to the use of this SCFA by colonocytes as an energy source [72,73].

The decrease in pH of the cecal contents of the PDX-supplemented groups (Sham PDX and GXT PDX) (Figure 3) represents an indicator of colonic fermentation and is in line with the increase in total SCFA production (Figure 4) observed in the present study.

There are several beneficial effects attributed to the fermentation of non-digestible carbohydrates in the colon and the production of SCFA. The literature reports that acidification of the colonic medium inhibits the growth of pathogenic bacteria, especially *Clostridium*, and indirectly increases the absorption of minerals, and reduces the absorption of ammonia [74]. Jie et al. [75], in a study with healthy Chinese subjects, evaluated the effect of beverages supplemented with 4, 8 or 12 g PDX on the physiological functions of the participants after 29 days. The authors observed several physiological benefits of PDX, including a decrease of the fecal pH. In addition, these authors also observed that the amount and type of SCFA in the feces of the individuals underwent major changes, especially a significant increase in butyric acid and acetic acid. Yoshioka; Shimomura and Suzuki [71] also observed a significant decrease in fecal pH after 92 days of experimentation in rats fed different concentrations of PDX, corroborating the results presented in this study.

The GXT groups (Control and PDX) showed a decline in HGB, HCT and MCV values shortly after surgery (time 0), an effect that was expected, since it has been reported by Santos et al. [39] that partial gastrectomy (Billroth II) with anterior truncal vagotomy is a good model to cause iron-deficiency anemia (hypochromic microcytic) in rats.

Throughout the supplementation period, HGB and HCT levels of the GXT PDX group remained higher than in the GXT Control group. These results suggest that PDX at 7.5% may have interfered with the alteration of the hematological profile of these animals, since according to Cançado and Chiattone [76], the initial stage for the recovery of iron deficiency anemia is the normalization of HGB and HCT levels. This takes around two months in humans, whose erythrocyte life span is 120 days, and the normalization of RBC indices occurs after two to six months of treatment. In rats, the erythrocyte life span is 60–65 days before being recycled into macrophages into organs such as the liver, spleen and bone marrow [77,78]. It could have required a longer treatment time for all the RBC indices to be normalized, since the HGB (29.2%) and HCT (55.8%) levels were significantly higher in GXT PDX animals.

The animals of the GXT groups (Control and PDX) had lower serum iron levels than the Sham groups (Control and PDX) (*p* < 0.0001). Santos et al. 2010 [10] also observed that gastrectomized animals had serum iron levels lower than in sham animals. It is noteworthy that when iron stores are depleted (as in the case of GXT animals in the present study), any further decline in body iron is accompanied by a reduction in serum iron concentration [79]. There was no statistical difference in serum iron concentrations between the GXT PDX and GXT Control groups (*p* = 0.7394). The same occurred with UIBC (*p* > 0.9999), TIBC (*p* = 0.9625) and TSI (*p* = 0.8044) values. However, it was observed that PDX triggered a slight improvement in serum iron parameters probably due to the reduction in the pH of the cecal contents and increase in the production of SCFA and cecum wall weight) in the supplemented animals, since serum iron concentration in the GXT PDX group was 35% higher when compared to the GXT Control group. However, iron deficiency caused by gastrectomy with anterior truncal vagotomy was not reversed. The animals of the Sham groups (PDX and Control) showed significantly higher TSI than the GXT (PDX and Control) groups (*p* < 0.0001). These results are in agreement with the reserve deficit of iron and serum iron shown by GXT animals in this study.

In a study with a similar experimental design, Santos et al. 2010 [10] evaluated the effect of 5% PDX supplementation for 8 weeks on iron absorption in rats with anemia induced by partial gastrectomy with anterior truncal vagotomy. The authors also observed that serum iron levels of PDX-fed animals (GXT and Sham) did not change (*p* > 0.05) compared to the control group (which did not receive PDX), corroborating the results of the present study. It should be pointed out that in the study mentioned, PDX was evaluated at a 5% concentration and in the present study the concentration was 7.5%, demonstrating that increasing the concentration of PDX did not lead to results different from those in the literature. Santos et al. 2010 [10] also observed that the HCT levels of GXT animals receiving PDX were higher compared to GXT animals fed a control diet, and did not differ from those of Sham rats (normals). HGB was also higher in the GXT rats supplemented with PDX, as seen in the present study.

Iron balance is a tightly controlled process that can be reflected in a number of iron status indicators and so the laboratory assessment of this mineral relies on a combination of biochemical indicators [80,81]. Serum ferritin (SF) represents a small fraction of the body’s ferritin pool, but the concentration of SF is reflective of the amount of iron stores. Once iron stores are depleted, the first stage of iron deficiency is reached, namely iron depletion, but there are no erythropoietic consequences yet [80]. At this time, serum iron concentration, TSI and HGB concentration remain normal and the measurement of SF concentration is one of the most sensitive methods for the diagnosis of this deficiency.

However, there are situations that can raise this indicator even in the presence of deficiency of this mineral, like when concomitant infection and inflammation are present due to ferritin’s action as an acute phase protein [82,83]. Therefore, this would not be a reliable indicator since GXT rats were susceptible to inflammatory conditions.

The major iron deposits in the body are located in the mononuclear phagocytic system: liver, spleen and bone marrow [84]. Hepatic iron concentration was lower in the GXT groups (Control and PDX) than in the Sham groups (Control and PDX) (*p* < 0.0001).

Lobo et al. [32] studied the influence of supplementation with fructooligosaccharides on iron uptake in anemic rats, and after 15 days, hepatic iron concentrations in anemic animals receiving prebiotic supplementation were similar to those in the control group. Takeuchi et al. [85] and Johnston et al. [86] stated that in anemia, there is an adaptive mechanism initially, in which the expression of genes regulating iron absorption is increased in both the duodenum and large intestine, which may explain the results presented here, since iron levels in the liver are parameters for the storage of this mineral, being a later stage of recovery from anemia.

In relation to the FPT expression values in the animals’ jejunum, cecum and colon there was no significant difference (*p* > 0.05) between any of the experimental groups; however, it was observed that the groups supplemented with PDX (Sham PDX and GXT PDX) showed a tendency for greater FPT expression in the jejunum and colon and that the GXT PDX group showed a tendency for greater FPT expression in the cecum in relation to the other experimental groups (Figure 7B). Marciano et al. [14] evaluated the effect of inulin (10%) and fructooligosaccharides (10%) supplementation on divalent metal transporter, FPT and cytochrome B in the duodenum, cecum and colon of anemic rats. After 2 weeks, they observed that none of the prebiotics produced a significant increase in these proteins in the duodenum. There was no increase in expression levels of FPT in the duodenum, cecum or proximal colon of the experimental groups, and it even decreased in the fructooligosaccharides group.

In another study with anemic rats conducted by Lobo et al. [32], the authors evaluated the influence of fructooligosaccharides on iron absorption and demonstrated that the expression of FPT in the cecum decreased in the groups that received prebiotics when compared to the control group, the authors suggested that the higher FPT expression in the control group may have occurred due to these animals still being anemic even after the treatment period.

These findings help explain the results obtained in the present study. The finding that the animals of the GXT Control group showed FPT expression in the jejunum, cecum and colon similar to that of the GXT PDX group can be explained by the fact that iron uptake is regulated by the animal’s need. In anemia, there is an adaptive mechanism in which an increase in the expression levels of genes that regulate iron absorption in the duodenum, cecum and colon [85,86]. This also suggests that PDX fermentation caused an increase in the expression of these genes, since the animals of the GXT PDX group had significantly higher HGB (29.2%) and HCT (55.8%) compared to the GXT Control group. This is in line with the notion that PDX is slowly fermented and that the main effects of its fermentation occur especially in the cecum and colon [61,71,87].

In relation to the FPT expression in the liver of the animals, the GXT PDX group showed the lowest concentration, differing statistically from the Sham Control group (*p* = 0,0482). Carvalho et al. [15] studied the effect of ingestion of partially GGPH on anemic rats and found a 368.3% increase in FPT expression in the cecum of the animals, but there was no significant difference in FPT expression in the duodenum and liver of the animals that received guar gum when compared to the control group.

It is known that hepcidin is a hormone essential for the regulation of iron homeostasis, and that it binds to FPT and regulates the exportation of iron to plasma [88]. However, the mechanism of FPT in enterocytes apparently differs from that of FPT in macrophages or hepatocytes [89,90].

In the study by Marciano et al. [14], cited above, the authors observed that the inulin and fructooligosaccharides groups did not alter serum and hepatic hepcidin concentrations compared to the control group. However, the fructooligosaccharides group showed a reduced urinary concentration of hepcidin, which was accompanied by a reduction in FPT protein expression in the duodenum. On the basis of these results, the authors suggested that there was an increase in the formation of the FPT-hepcidin complex, in which FPT is internalized and degraded, leading them to question the veracity of this pattern in reducing iron absorption and increasing anemia, since these animals showed HGB and HCT concentrations similar to those of the other groups, as seen in the present study.

On the basis of the results reported by Marciano et al. [14], it is possible to infer that there was an increase in the formation of the FPT-hepcidin complex in the GXT animals (Control and PDX) in the present study. This might have occurred because of the possible triggering of inflammatory conditions. It is believed that after the gastrectomy, the region of the anastomosis starts to show chronic proinflammatory characteristics due to the presence of foreign bodies (sutures) and the continuous contact with the duodenogastric contents [91]. It is known, therefore, that hepcidin production is stimulated in vivo and in vitro by LPS or turpentine, standard inflammatory stimuli. This stimulation appears to be mediated by the inflammatory cytokine IL-6 [92].

Thus, the lower expression of hepatic FPT in the GXT groups (Control and PDX) in relation to the Sham Control group can be explained by the formation of the FPT-hepcidin complex, resulting from the possible inflammatory picture generated by the gastric resection surgery. However, this did not prevent the improvement in HGB (29.2%), HCT (55.8%) and serum iron (35%) observed in the animals of the GXT PDX group.

Pak et al. [93] found that anemia does not directly regulate the expression of hepcidin, but it does exert its effects on this hormone and iron metabolism through a substance not yet identified, released during erythropoiesis. The specific mediators and pathways by which they influence the synthesis and release of hepcidin need to be elucidated.

Considering the lower FPT expression in the liver of the animals of the GXT PDX group in the present study, we can suppose that due to the high degree of polymerization of the PDX molecule, its metabolites exert a greater influence on the physiological modifications in the large intestine and that FPT is responsible for the efflux of intracellular iron. Perhaps this prebiotic is more directly related to the increased expression of receptors related to the promotion of iron absorption. Further studies are needed to evaluate the effect of PDX on other iron absorption and storage receptors to elucidate and establish the effectiveness of PDX.

The 7.5% PDX demonstrated potential effects for the improvement of anemia and increased production of SCFA in post-gastrectomy rats, and the large intestine is involved in the iron absorption process. Many of these physiological benefits are interrelated and SCFA plays a central role. A longer treatment time may be necessary for better absorption of iron and consequently normalization of all RBC indices. Finally, while there is emerging evidence for increased nutrient uptake through the production of SCFA, observed here through PDX supplementation, further studies are needed, especially in humans.

## 5. Conclusions

The data showed that the lower dietary intake by the GXT PDX group did not influence the animals’ body weight gain and body development. The slow and gradual fermentation of PDX produced morphological and physiological changes in the cecum of the supplemented animals, where there was a decrease in pH, weight increase and marked production of SCFA, especially acetic acid and propionic acid. Levels of serum iron, TSI, MCV and MCHC were lower, and those of UIBC and TIBC were higher in the GXT groups than in the Sham groups. HGB and HCT values were higher in the groups receiving PDX supplementation. Fresh liver weights did not differ between groups during the experiment. Hepatic iron levels were lower in GXT animals. Gastrectomy and PDX supplementation did not alter the expression of FPT in the animals’ jejunum, cecum and colon. The GXT PDX animals showed lower expression of hepatic FPT. PDX led to increased SCFA concentration in the supplemented animals. Considering that SCFAs play a central role in increasing nutrients uptake, this mechanism may be involved in altering the hematology profile observed in these animals but not enough to reverse iron deficiency anemia in post-gastrectomy rats. Perhaps, a longer treatment time is necessary to see better absorption of iron and consequently normalization of all RBC indices.

## Figures and Tables

**Table 1:** Composition of experimental diets (g/kg) in accordance with AIN-93M [38].

Ingredient	Control Diet *	Supplemented Diet (PDX) **
Cornstarch ^2^	466	441
Maltodextrin ^3^	155	155
Casein ^3^^,^***	140	140
Sucrose ^4^	100	100
Soybean oil ^5^	40	40
Cellulose ^3^	50	0
**Polydextrose** ^1^	**0**	**75**
Mineral mix ^3^	35	35
Vitamin mix ^3^	10	10
L-cystine ^6^	1.8	1.8
Choline bitartrate ^7^	2.5	2.5
Tert-butylhydroquinone ^7^	0.008	0.008

¹ Commercial and Industrial Development Ecil Ltda, São Paulo, SP, Brazil; ^2^ Emifor, MG, Brazil; ^3^ Rhoster Indústria e Comércio Ltda, Araçoiaba da Serra, SP, Brazil; ^4^ Camil Foods S/A, Araquari, SC, Brazil; ^5^ Bunge Foods S.A, Gaspar, SC, Brazil; ^6^ Labsynth Products for Laboratories Ltda. Diadema, SP, Brazil; ^7^ Sigma-Aldrich Brazil Ltda, São Paulo, Brazil; *** Crude protein >85%; * No addition of polydextrose; ** With addition of polydextrose (7.5%).

**Table 2:** Composition, iron and energy value of experimental diets formulated according to AIN-93M.

Chemical Component	Without Polydextrose (AIN-93M Standard)	With Polydextrose (7.5%)	*p*-Value
Total solids	90.12 ± 0.26 ^b^	90.89 ± 0.36 ^a^	0.0139
Moisture (%)	9.11 ± 0.36 ^b^	9.88 ± 0.26 ^a^	0.0491
Total ash (g/100 g)	3.03 ± 0.02 ^b^	2.81 ± 0.05 ^a^	0.0002
Lipids (g/100 g)	4.89 ± 0.90 ^a^	4.89 ± 0.71 ^a^	0.9975
Proteins (g/100 g)	9.55 ± 0.24 ^a^	9.52 ± 0.30 ^a^	0.8748
Total carbohydrates (g/100 g) *	73.42 ± 1.36 ^a^	72.91 ± 0.50 ^a^	0.5086
Iron (mg/g)	0.12 ± 0.02 ^a^	0.12 ± 0.02 ^a^	0.9625
Caloric value (kcal/100 g) ***	375.87 ± 3.18 ^a^	373.69 ± 3.72 ^a^	0.4076

Values expressed as mean ± standard deviation. Same letters in the same row indicate no significant difference according to Student’s *t*-test (*p* > 0.05). Different letters in the same row indicate significant difference according to Student’s *t*-test (*p* < 0.05). * Determination of total carbohydrates by difference. *** Calculated by sum: (crude protein × 4) + (total lipids × 9) + (carbohydrates × 4).

**Table 3:** Dietary intake, weight, body length and feed efficiency coefficient (FEC) of the animals in the respective experimental groups at the end of the supplementation period.

Variable	Sham Control	Sham PDX	GXT Control	GXT PDX	*p*-Value
Dietary intake (g)	1149.45 ± 5.81 ^a^	1070.96 ± 6.69 ^b^	1087.84 ± 5.46 ^b^	1008.88 ± 5.81 ^c^	<0.0001
Body weight (g)	148.00 ± 10.56 ^a^	137.70 ± 6.29 ^a^	94.2 ± 9.11 ^b^	116.60 ± 16.25 ^a,b^	0.0067
Body length (cm)	24.19 ± 0.62 ^a^	23.88 ± 069 ^a^	23.79 ± 0.31 ^a^	23.93 ± 1.14 ^a^	0.8091
FEC	0.13 ± 0.01 ^a^	0.13 ± 0.01 ^a^	0.09 ± 0.01 ^b,c^	0.12 ± 0.02 ^b^	0.0007

Sham Control: sham without polydextrose; Sham PDX: sham with polydextrose (7.5%); GXT Control: gastrectomized without polydextrose; GXT PDX: gastrectomized with polydextrose (7.5%). FE = feed efficiency. Values expressed as mean ± SEM. ANOVA followed by Tukey’s post-test. Same letters in the same row indicate no significant difference according to Tukey’s test (*p* > 0.05). Different letters in the same row indicate a significant difference according to Tukey’s test (*p* < 0.05).

**Table 4:** Serum iron, UIBC, TIBC and TSI levels of the animals of the experimental groups.

Variable	Sham Control	Sham PDX	GXT Control	GXT PDX	*p*-Value
Serum iron (µg/dL)	160.25 ± 44.62 ^a^	167.00 ± 24.86 ^a^	37.78 ± 20.77 ^b^	51.00 ± 20.67 ^b^	<0.0001
UIBC (µg/dL)	317.00 ± 50.58 ^b^	304.00 ± 70.39 ^b^	618.67 ± 43.64 ^a^	618.57 ± 63.02 ^a^	<0.0001
TIBC (µg/dL)	477.25 ± 60.19 ^b^	479.30 ± 14.90 ^b^	654.17 ± 52.25 ^a^	667.38 ± 66.27 ^a^	<0.0001
TSI (%) *	32.88 ± 9.85 ^a^	33.70 ± 8.23 ^a^	4.83 ± 5.24 ^b^	7.25 ± 5.38 ^b^	<0.0001

Values expressed as mean ± SEM. ANOVA followed by Tukey’s post-test. Sham Control: sham without polydextrose; Sham PDX: sham with polydextrose (7.5%); GXT Control: gastrectomized without polydextrose; GXT PDX: gastrectomized with polydextrose (7.5%). * TSI: transferrin saturation index. Different letters in the same row indicate a significant difference according to Tukey’s test (*p* < 0.05).
